# Central Responses to SGLT2 Inhibition: Distinguishing Direct CNS Pharmacology from the Brain–Kidney–Metabolism Axis

**DOI:** 10.3390/ijms27219514

**Published:** 2026-10-24

**Authors:** Dong Hee Kim, Min Jin Lee, Seung Hee Lee, Joo Yeon Kim, Ji Hyun Bae, Ah Reum Khang, Yang Ho Kang, Dongwon Yi

**Affiliations:** 1 Research Institute for Convergence of Biomedical Science and Technology, Pusan National University Yangsan Hospital, Yangsan 50612, Republic of Korea; gainlydh@pusan.ac.kr (D.H.K.); minjinpig@hanmail.net (M.J.L.); shl8148@gmail.com (S.H.L.); rlawndus0226@naver.com (J.Y.K.); yaho1229@gmail.com (J.H.B.); medikar82@gmail.com (A.R.K.); kangyh@pusan.ac.kr (Y.H.K.); 2 Division of Endocrinology and Metabolism, Department of Internal Medicine, Pusan National University Yangsan Hospital, Pusan National University School of Medicine, Yangsan 50612, Republic of Korea

**Keywords:** sodium-glucose transporter 2 inhibitors, hypothalamus, energy metabolism, autonomic nervous system, ketone bodies, neuroinflammatory diseases, kidney

## Abstract

Sodium-glucose cotransporter 2 (SGLT2) inhibitors improve cardiovascular and kidney outcomes through mechanisms that extend beyond glycemic control. Emerging studies report changes in hypothalamic and brainstem physiology following SGLT2 inhibitor administration, but the evidentiary meaning of these observations remains uncertain. This review critically evaluates clinical, in vivo, ex vivo, and cellular evidence linking SGLT2 inhibition to central metabolic regulation. We distinguish three claims that are often conflated: central pharmacological capacity after direct neural exposure, central nervous system (CNS) adaptation after systemic treatment, and therapeutically relevant CNS target engagement with causal mediation. Candidate pathways include hypothalamic nutrient sensing, insulin responsiveness, melanocortin signaling, ketone availability, glial inflammation, redox regulation, and autonomic activity. We propose a bidirectional brain–kidney–metabolism framework in which renal glucose and sodium loss generate metabolic, hormonal, inflammatory, hemodynamic, and potentially neural signals that are integrated by hypothalamic and brainstem circuits. These circuits may then convey autonomic and neuroendocrine outputs to peripheral organs. Current evidence supports central responsiveness and CNS adaptation to systemic treatment, but not a unified direct SGLT2-dependent brain mechanism. Compound-specific unbound CNS pharmacokinetics, orthogonal cell-specific target validation, compartment-restricted models, selective renal-afferent manipulation, and longitudinal human phenotyping are required to establish causality. The proposed framework complements, rather than replaces, established renal and tissue-specific mechanisms.

## 1. Introduction

Sodium-glucose cotransporter 2 (SGLT2) inhibitors were developed to reduce plasma glucose through the inhibition of glucose and sodium reabsorption in the renal proximal tubule. Large cardiovascular and kidney outcome trials subsequently demonstrated protection against heart failure events and chronic kidney disease progression, including in individuals without diabetes, for whom glucose lowering cannot fully explain the observed benefits [1,2,3,4]. Additional trials across the heart failure spectrum established that these effects are not restricted to patients with reduced ejection fraction or concomitant diabetes [5,6]. These findings repositioned SGLT2 inhibitors as therapies that induce broad cardiorenal and metabolic adaptation. Their effects arise from interacting renal hemodynamic, energetic, endocrine, inflammatory, and interorgan processes rather than a single downstream pathway [7,8]. Consistent with this concept, experimental metabolic-tracing and systems-level analyses have demonstrated coordinated reprogramming of glucose oxidation, redox metabolism, and energy-sensing pathways across the kidney and distant organs after SGLT2 inhibition [9]. Moreover, renal glucosuria induces changes in insulin and glucagon concentrations, lipid oxidation, ketogenesis, and compensatory energy balance, thereby generating multiple potential signals by which a primarily renal intervention may influence the central nervous system (CNS) [10,11]. The CNS is a plausible, though incompletely resolved, component of this response. The hypothalamus and interconnected brainstem nuclei integrate nutrient availability, adiposity, hormonal state, visceral signals, and cellular energy status into behavioral, neuroendocrine, and autonomic outputs that regulate peripheral glucose and energy metabolism [12,13]. Experimental SGLT2 inhibition has been linked to changes in feeding-related signaling, hypothalamic insulin responsiveness, glial inflammation, and brainstem neuronal activity [14,15,16,17,18,19,20]. A CNS phenotype after systemic treatment, however, does not demonstrate direct CNS SGLT2 engagement because renal glucosuria simultaneously alters glucose, insulin, glucagon, ketone bodies, adiposity-related hormones, inflammatory tone, volume status, and potentially renal afferent signaling [11,21]. Although SGLT2/*SLC5A2* or SGLT2/*Slc5a2* immunoreactivity and transcript expression have been reported in choroid plexus epithelial and ependymal cells, these observations do not establish functional expression in hypothalamic neurons or demonstrate target engagement at therapeutic unbound drug concentrations [22]. Accordingly, this review distinguishes evidence of central pharmacological capacity from evidence of therapeutic CNS target engagement and evaluates the hypothalamus within an integrated brain–kidney–metabolism axis.

## 2. Physiological Architecture of Hypothalamic Energy Homeostasis

### 2.1. The Arcuate Nucleus as a Metabolic Sensing Hub

**Figure 1:**
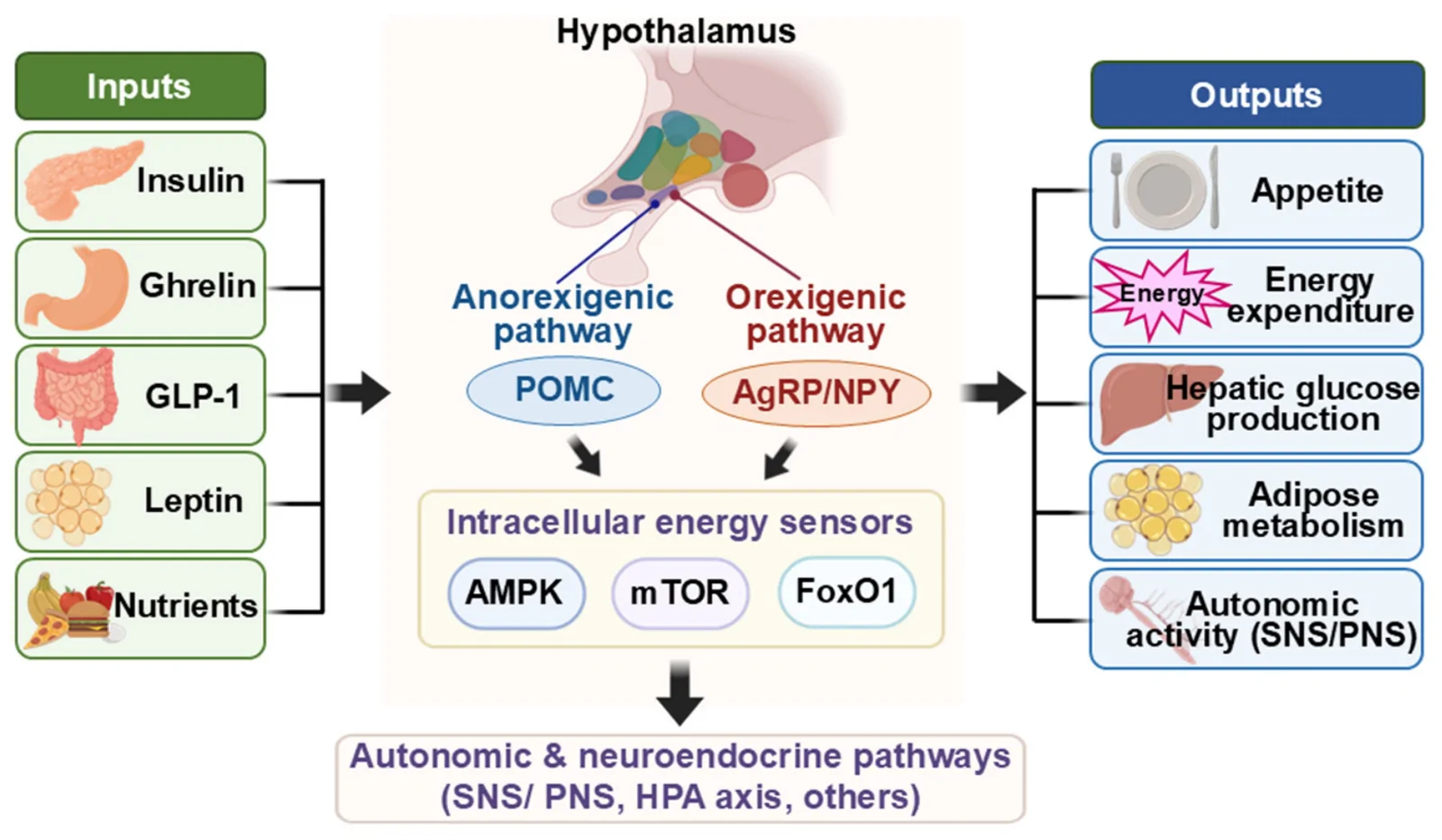
**Physiological organization of hypothalamic energy sensing and systemic metabolic regulation.** The hypothalamus integrates circulating hormonal and nutrient inputs, including insulin, ghrelin, glucagon-like peptide-1 (GLP-1), leptin, and nutrients, to regulate whole-body energy homeostasis. Within the arcuate nucleus, the orexigenic agouti-related peptide/neuropeptide Y (AgRP/NPY) pathway and the anorexigenic pro-opiomelanocortin (POMC) pathway process these signals through intracellular energy sensors, including AMP-activated protein kinase (AMPK), mechanistic target of rapamycin (mTOR), and forkhead box O1 (FoxO1). These sensors converge on autonomic and neuroendocrine output pathways (sympathetic nervous system activity, parasympathetic nervous system (PNS) activity, the hypothalamic–pituitary–adrenal (HPA) axis, and related circuits), which regulate appetite, energy expenditure, hepatic glucose production, adipose tissue metabolism, and autonomic activity. Additional contributors to this circuitry, including astrocytes, microglia, tanycytes, and phosphoinositide 3-kinase-Akt signaling, are described in the main text (Section 2.1 and Section 2.3) but are not individually depicted in this schematic.

The mediobasal hypothalamus is anatomically situated to sense circulating metabolic information and coordinate adaptive responses. Within the arcuate nucleus (ARC), agouti-related peptide/neuropeptide Y (AgRP/NPY) neurons are activated during fasting and negative energy balance and promote food-seeking behavior and reduce selected energy-expending processes [23,24,25]. By contrast, pro-opiomelanocortin (POMC) neurons contribute to melanocortin signaling through the release of α-melanocyte-stimulating hormone and the activation of melanocortin-4 receptors in downstream nuclei, including the paraventricular hypothalamus [26,27]. The functional output of these circuits extends beyond feeding behavior and includes glucose production, insulin action, adipose metabolism, and autonomic regulation [13,28]. Nevertheless, the classical dichotomy between orexigenic AgRP/NPY and anorexigenic POMC neurons remains useful but incomplete because these populations are transcriptionally, electrophysiologically, and projectionally heterogeneous and participate in temporally distinct components of feeding and metabolic regulation [29,30]. ARC neurons also interact with astrocytes, microglia, tanycytes, endothelial cells, and perivascular cells. Together, these cellular populations contribute to glucose sensing and transport, hormonal access, inflammatory signaling, synaptic remodeling, and the maintenance of the local metabolic microenvironment [31,32,33,34,35]. In metabolic disease, these cellular interactions may undergo state-dependent remodeling, with glial and neurovascular responses either supporting adaptive nutrient sensing or contributing to chronic hypothalamic inflammation and neuronal dysfunction. Figure 1 schematically summarizes this physiological architecture.

### 2.2. Peripheral Hormonal and Nutrient Inputs

Circulating hormones, nutrients, and gut-derived signals provide hypothalamic and brainstem circuits with complementary information about long-term energy stores, current metabolic status, and recent nutrient intake. Leptin functions primarily as an adiposity-related signal, whereas insulin reflects both adiposity and nutrient availability. These hormones act on distributed neuronal and glial populations, including AgRP/NPY and POMC neurons, among others, to influence feeding, energy expenditure, glucose homeostasis, and autonomic output [12,36,37]. By contrast, ghrelin rises during fasting and before meals and promotes hunger partly by activating hypothalamic AgRP/NPY pathways [38,39]. Glucose, fatty acids, amino acids, and their intracellular metabolites are also detected by specialized neuronal and glial sensing systems, allowing central circuits to respond directly to changes in substrate availability. Postprandial gastrointestinal signals add another layer of regulation. Glucagon-like peptide-1 (GLP-1), peptide YY (PYY), cholecystokinin, and nutrient-derived signals engage vagal afferents, brainstem circuits, and, in a context-dependent manner, central receptors to regulate satiation, gastric emptying, pancreatic hormone secretion, and glucose metabolism [40,41,42,43]. The relative contributions of vagal and direct central pathways depend on the peptide, its site of release, and whether the signal is endogenous or pharmacologically administered. Integration of these hormonal, nutrient, and neural inputs affects food seeking and meal termination as well as hepatic glucose production, pancreatic function, adipose tissue metabolism, and peripheral glucose disposal through neuroendocrine and autonomic pathways [13,44]. This physiological framework is particularly relevant to SGLT2 inhibition because renal glucosuria causes a persistent loss of glucose-derived energy while simultaneously altering glycemia, insulin and glucagon concentrations, lipid oxidation, ketone availability, body composition, and fluid and sodium balance. Thus, unlike centrally acting anti-obesity agents whose primary pharmacological objective is appetite suppression, SGLT2 inhibitors initiate a renal metabolic perturbation that may elicit homeostatic responses at both the peripheral and central levels. Mathematical modeling and some experimental studies suggest that increased energy intake can partially offset urinary calorie loss [11]. However, randomized human studies have not consistently shown increases in appetite, measured food intake, energy expenditure, or food-cue-related brain activity during SGLT2 inhibitor treatment [20,45,46]. Compensatory feeding should therefore be considered a context-dependent and time-dependent possibility rather than an established class-wide response.

### 2.3. Intracellular Energy Sensing

Hypothalamic neurons and glial cells translate changes in nutrient and hormonal availability into cellular and circuit-level responses through interconnected signaling networks that include AMP-activated protein kinase (AMPK), mechanistic target of rapamycin complex 1 (mTORC1), phosphoinositide 3-kinase (PI3K)–Akt signaling, forkhead box O1 (FoxO1), mitochondrial dynamics, and redox-sensitive pathways. Rather than functioning as independent sensors, these pathways interact to regulate membrane excitability, neuropeptide expression, synaptic organization, cellular substrate utilization, and neuroendocrine and autonomic output [37,47,48,49]. AMPK is activated by an increase in the AMP/ATP or ADP/ATP ratio and by upstream kinases, and therefore generally signals reduced cellular energy availability. In several hypothalamic regions, increased AMPK activity during fasting or glucoprivation promotes feeding, whereas leptin, insulin, glucose, and refeeding generally reduce hypothalamic AMPK activity [47,50]. However, these effects depend on neuronal population, hypothalamic nucleus, duration of activation, and metabolic context. Conversely, hypothalamic mTORC1 responds to amino acids, growth factors, and energy sufficiency. Leucine and leptin can activate hypothalamic mTORC1 signaling, and pharmacological inhibition of this pathway can attenuate their anorectic effects [48]. Chronic or inappropriate mTORC1 activation, however, may impair neuronal responsiveness and promote feedback inhibition of insulin signaling, indicating that mTORC1 activity should not be interpreted as a simple linear marker of satiety. Insulin and leptin engage partially overlapping but nonidentical intracellular pathways in hypothalamic neurons. Insulin receptor activation recruits insulin receptor substrates and PI3K–Akt signaling, whereas leptin acts primarily through Janus kinase 2-signal transducer and activator of transcription 3 signaling and can also influence the PI3K–Akt and AMPK pathways. In the canonical PI3K–Akt–FoxO1 pathway, Akt-dependent phosphorylation promotes nuclear exclusion of FoxO1. Reduced nuclear FoxO1 activity suppresses orexigenic AgRP/NPY transcription while relieving FoxO1-mediated repression of POMC, thereby favoring melanocortin signaling [37,51,52]. These transcriptional effects are accompanied by rapid changes in ion-channel activity and neuronal excitability and should therefore not be interpreted as the sole mechanism through which insulin and leptin regulate AgRP and POMC neurons. Mitochondrial substrate oxidation, reactive oxygen species generation, antioxidant capacity, and mitochondrial fission and fusion add another layer of metabolic sensing. Nutrient-dependent remodeling of mitochondrial morphology and function has been implicated in the regulation of both AgRP and POMC neuronal activity [53,54,55]. Reactive oxygen species can function as physiological signaling intermediates, but persistent oxidative stress may disrupt hypothalamic insulin and leptin responsiveness, induce inflammatory signaling, and impair neuronal function. Thus, measurements of oxidative stress or mitochondrial abundance alone cannot establish whether a metabolic pathway is activated adaptively or pathologically. These intracellular networks provide plausible interfaces by which the systemic consequences of SGLT2 inhibition, including urinary energy loss, reduced insulin concentrations, an increased glucagon-to-insulin ratio, enhanced lipid oxidation, ketone production, and changes in inflammatory and redox states, could influence hypothalamic function. Limited experimental evidence indicates that the intracerebroventricular administration of SGLT2 inhibitors can increase hypothalamic AMPK phosphorylation and food intake [14]. However, modulation of AMPK, Akt, FoxO1, mTORC1, mitochondrial function, or inflammatory signaling after systemic treatment does not establish direct CNS target engagement. These changes may instead result from altered circulating nutrients, hormones, ketones, cytokines, or energy balance. In addition, some SGLT2 inhibitors can alter cellular energetics through SGLT2-independent mechanisms; canagliflozin, for example, activates AMPK in cultured cells by inhibiting mitochondrial respiratory-chain complex I at high concentrations [56]. Establishing a direct central mechanism therefore requires evidence that unbound drug concentrations in the relevant CNS compartment are sufficient for target inhibition, together with cell-specific target validation and pharmacological or genetic demonstration of SGLT2 dependence.

### 2.4. Autonomic and Neuroendocrine Outputs

Hypothalamic metabolic circuits influence peripheral physiology through distributed autonomic and neuroendocrine pathways. Neurons in the paraventricular, dorsomedial, ventromedial, arcuate, and lateral hypothalamic regions communicate with brainstem autonomic nuclei, including the nucleus tractus solitarius (NTS), dorsal motor nucleus of the vagus, and rostral ventrolateral medulla (RVLM). Some hypothalamic neurons project directly to sympathetic preganglionic neurons in the intermediolateral cell column of the spinal cord, whereas others modulate sympathetic and parasympathetic outflow indirectly through brainstem relays [12,13]. The NTS integrates vagal and other visceral afferent information, while the RVLM contains premotor neurons that provide major excitatory drive to spinal sympathetic circuits involved in cardiovascular and renal regulation. Through these pathways, hypothalamic and brainstem networks regulate hepatic glucose production, pancreatic insulin and glucagon secretion, white adipose tissue lipolysis, brown adipose tissue thermogenesis, skeletal muscle glucose uptake, cardiovascular function, and renal physiology [13,57,58,59,60,61]. Specifically, melanocortin-4 receptors expressed in autonomic neurons contribute to the regulation of sympathetic and parasympathetic activity, thermogenesis, and glycemia [59,60]. In the kidney, efferent renal sympathetic nerves influence renin release, tubular sodium reabsorption, renal vascular resistance, and glomerular hemodynamics. Conversely, renal sensory afferents detect mechanical and chemical changes within the kidney and renal pelvis and convey this information to spinal and supraspinal autonomic networks. These afferent signals participate in renorenal reflexes, and particularly under pathological conditions, may contribute to sustained central sympathetic activation [21,62]. Hypothalamic neurons also regulate endocrine physiology through releasing and inhibiting hormones delivered to the anterior pituitary and through direct projections to neurosecretory neurons controlling vasopressin and oxytocin release. Consequently, metabolic signals integrated by the hypothalamus can influence the hypothalamic–pituitary–adrenal, hypothalamic–pituitary–thyroid, reproductive, and fluid–balance axes. These neuroendocrine responses interact with autonomic outputs rather than forming an entirely separate regulatory system. This organization provides a physiological basis for bidirectional brain–kidney communication during SGLT2 inhibition. Renal glucosuria, natriuresis, osmotic diuresis, and changes in renal metabolism may generate circulating metabolic, hormonal, hemodynamic, inflammatory, and potentially renal afferent neural signals. Once integrated by hypothalamic and brainstem networks, these signals could modify sympathetic, parasympathetic, and neuroendocrine outputs to the kidney and other metabolic organs. Experimental and clinical studies suggest that SGLT2 inhibition may modulate autonomic balance or attenuate the sympathetic activation expected from natriuresis and volume loss; however, direct microneurographic studies have not demonstrated a uniform reduction in resting muscle sympathetic nerve activity [19,63,64,65,66]. It remains uncertain whether these responses result from direct drug action within central autonomic nuclei, altered renal afferent signaling, changes in volume and sodium balance, improved metabolic status, or a combination of these mechanisms. The proposed brain–kidney–metabolism feedback loop is therefore best regarded as a testable integrative framework rather than an established mechanism of SGLT2 inhibitor action.

## 3. Renal SGLT2 Inhibition as a Systemic Metabolic Perturbation

The primary pharmacological action of current SGLT2 inhibitors occurs in the early renal proximal tubule. Reduced glucose and sodium reabsorption produces glucosuria, natriuresis, and osmotic diuresis, increases distal sodium delivery, and modifies tubuloglomerular feedback and intraglomerular hemodynamics while reducing proximal tubular transport workload and systemic carbohydrate availability [7,67,68,69]. Although afferent arteriolar vasoconstriction provides an important mechanism in hyperfiltering type 1 diabetes, studies in type 2 diabetes indicate that post-glomerular vasodilation may also contribute; thus, the renal hemodynamic response should not be reduced to a single vascular mechanism [68,69]. Multiorgan metabolomic and proteomic studies further show that renal SGLT2 inhibition changes circulating and extrarenal metabolic networks, which supports pharmacologically induced interorgan communication rather than an isolated renal effect [8,9]. In humans, glucosuria is accompanied by increased endogenous glucose production, changes in insulin sensitivity and β-cell workload, and a shift from carbohydrate toward lipid oxidation [10,70,71,72,73]. A reproducible adaptation is a shift toward lipid mobilization and hepatic ketogenesis, driven partly by lower insulin exposure and a relative increase in glucagon signaling [10,70,71,73]. The contribution of direct pancreatic α-cell SGLT2 signaling remains controversial, and changes in the glucagon-to-insulin ratio are therefore better interpreted as an integrated systemic response rather than unequivocal evidence of direct α-cell target engagement. β-Hydroxybutyrate can function as both an oxidative substrate and a signaling metabolite that influences redox balance, protein modification, and inflammatory pathways [74,75]. Because circulating ketone bodies cross the blood–brain barrier and can be used by neurons and glia, SGLT2 inhibitor-associated ketogenesis provides a plausible indirect route by which renal energy loss could alter the cerebral metabolic environment. However, increased ketone availability does not establish a requirement for ketone oxidation or signaling for hypothalamic, cardiovascular, or renal benefit. Ketosis should therefore be considered a candidate mediator rather than an established causal mechanism [76]. Endocrine and behavioral adaptations may also be time dependent. Urinary calorie loss may elicit early compensatory responses, whereas chronic treatment can lead to a new metabolic steady state in which measured appetite and food intake are not persistently increased [11,46]. The discrepancy between predicted weight loss based on urinary energy excretion and observed weight loss is consistent with compensatory energy intake, but mathematical inference should be distinguished from the direct measurement of appetite or caloric consumption [11]. This renal perturbation provides the mechanistic context for evaluating whether subsequent CNS changes reflect direct drug action or secondary adaptation.

## 4. Does SGLT2 Inhibition Directly Affect the Brain?

### 4.1. Defining Evidence for a Direct CNS Mechanism

**Figure 2:**
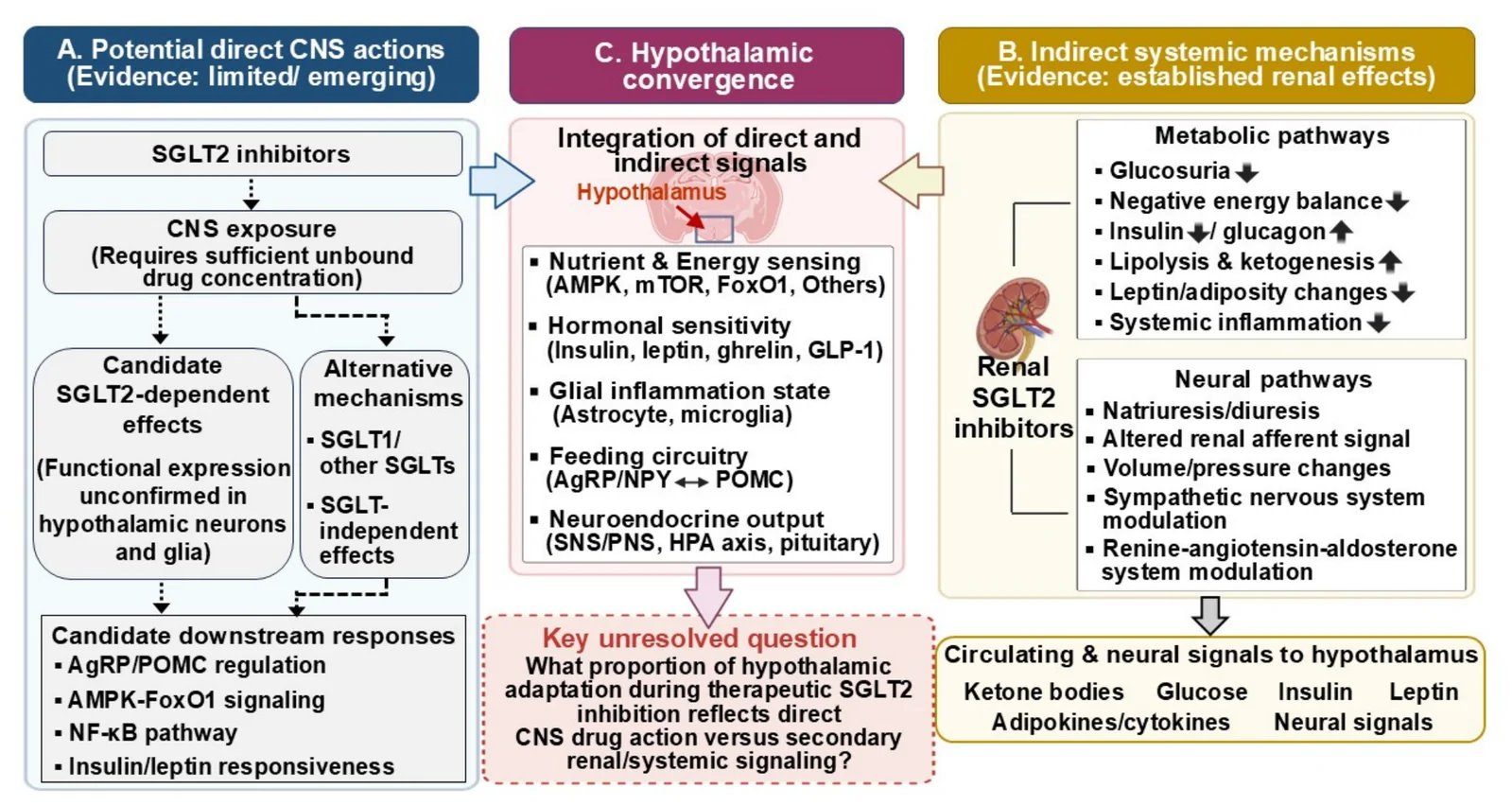
**Evidence framework for direct and indirect effects of SGLT2 inhibition on central metabolic regulation.** Sodium-glucose cotransporter 2 (SGLT2) inhibition may influence hypothalamic and brainstem function through distinct but potentially convergent pathways, summarized in three linked panels. (**A**) Proposed direct CNS actions require sufficient unbound drug exposure and a functionally validated cellular target. SGLT1/other SGLTs and SGLT-independent mechanisms are shown as alternative explanations. Dashed arrows indicate proposed relationships; functional SGLT2 expression in hypothalamic neurons and glial cells and SGLT2-specific CNS target engagement during therapeutic oral dosing remain insufficiently established. (**B**) Indirect mechanisms originate predominantly from renal SGLT2 inhibition and include glucosuria, natriuresis, negative energy balance, altered insulin-to-glucagon signaling, enhanced lipid mobilization and ketogenesis, changes in adiposity-related hormones, reduced systemic inflammatory stress, altered volume and pressure loading, and potentially modified renal afferent signaling. (**C**) Circulating metabolites, hormones, inflammatory mediators, and neural inputs may converge on hypothalamic and brainstem circuits and modify nutrient sensing, glial activation, feeding behavior, and autonomic and neuroendocrine output. Solid lines indicate pathways supported by relatively direct experimental evidence, whereas dashed or dotted lines indicate indirect, associative, or proposed relationships for which causal evidence remains limited.

Establishing a direct CNS mechanism requires stronger evidence than demonstration of a CNS-associated phenotype after systemic SGLT2 inhibitor treatment. Such a phenotype may arise from direct drug action within the brain, but it may also reflect secondary responses to renal glucosuria, altered circulating metabolites and hormones, changes in volume or sodium balance, reduced systemic inflammation, or renal afferent neural signaling. Evidence of brain exposure, local pharmacological activity, and molecular target engagement must therefore be considered separately. Evidence for a direct CNS mechanism would ideally include several complementary lines of evidence. First, the proposed molecular target should be reproducibly detected in the relevant CNS cell type using methods with adequate transcript-level, protein-level, and cellular specificity. SGLT2/*SLC5A2* or SGLT2/*Slc5a2* immunoreactivity has been reported in choroid plexus epithelial and ependymal cells in human and mouse brains, together with *Slc5a2* transcript detection in mouse choroid plexus [22]. However, these findings do not establish functional transporter activity in hypothalamic neurons or demonstrate that the same cellular distribution is conserved across species, developmental stages, and metabolic conditions. Second, the detected target should be shown to function as a sodium-glucose transporter in that cell population. Third, after clinically relevant systemic dosing, pharmacologically active unbound drug concentrations should reach the corresponding extracellular or intracellular CNS compartment. Measurements of total drug in whole-brain homogenates are insufficient because they can reflect residual vascular drug, nonspecific tissue binding, or accumulation in brain barriers rather than exposure at the cellular site of action [77,78]. Ideally, unbound brain exposure should be compared with the unbound concentration of the administered SGLT2 inhibitor required to inhibit the relevant transporter under physiological sodium and glucose conditions. Fourth, localized CNS exposure should reproduce the biological response in the absence of renal glucosuria or other systemic renal effects. Fifth, genetic deletion or selective blockade of CNS SGLT2 should abolish the response, whereas selective blockade of renal SGLT2 should not abolish it, provided that adequate CNS drug exposure is maintained. Kidney-restricted inhibitors, CNS-restricted delivery, cell-specific *Slc5a2* deletion, and rescue experiments would be especially informative. Finally, temporal and dose–response concordance among CNS exposure, target inhibition, intracellular signaling, neural activity, and physiological outcome would support causal inference. Most available studies satisfy only one or a few of these criteria. Current evidence therefore supports the capacity of sodium-glucose cotransporter (SGLT) inhibitors to alter CNS physiology under selected experimental conditions but does not establish that the direct inhibition of hypothalamic SGLT2 contributes meaningfully to the systemic effects of therapeutic oral administration. This distinction is especially important because sodium-coupled glucose sensing in the hypothalamus predates the development of selective SGLT2 inhibitors and has been linked experimentally to sodium-glucose cotransporter 1 (SGLT1) and sodium-glucose cotransporter 3 rather than specifically to SGLT2 [79,80]. Accordingly, Figure 2 distinguishes unconfirmed SGLT2-dependent CNS effects from possible SGLT1/other SGLT-mediated and SGLT-independent mechanisms.

### 4.2. Central Administration and Hypothalamic Cellular Studies

Earlier studies of intracerebroventricular phlorizin demonstrated Fos induction in ependymal cells, periventricular glia, and neurons located in the ARC, paraventricular hypothalamus (PVH), ventromedial hypothalamus (VMH), and NTS [81]. Because phlorizin lacks the isoform selectivity of contemporary gliflozins, these observations support a central SGLT-sensitive mechanism but do not implicate SGLT2 specifically. Takeda and colleagues provided proof-of-principle evidence that centrally delivered SGLT inhibitors can influence feeding behavior. Intracerebroventricular administration of tofogliflozin, dapagliflozin, or empagliflozin increased acute food intake in healthy rats, whereas the tested peripheral administration protocol produced no comparable response. Tofogliflozin also increased AMPK phosphorylation and c-Fos immunoreactivity in the lateral hypothalamic area [14]. These findings show that direct exposure of CNS compartments to SGLT inhibitors can engage hypothalamic signaling associated with feeding. Nevertheless, this study requires cautious interpretation. Intracerebroventricular dosing bypasses the blood–brain barrier and blood–cerebrospinal fluid barrier and can expose ventricular, periventricular, and hypothalamic structures to concentrations and concentration–time profiles that differ substantially from those achieved after oral treatment. A negative response to peripheral administration at a nominally similar dose does not establish equivalent exposure at the neural target. In addition, activation of AMPK or c-Fos does not identify which molecular transporter is responsible for the response. These outcomes could reflect the inhibition of SGLT2, partial inhibition of SGLT1, changes in local glucose handling, or transporter-independent cellular stress. Pharmacological differences limit extrapolation across SGLT2 inhibitors. Empagliflozin and tofogliflozin show high SGLT2 selectivity over SGLT1, whereas dapagliflozin is less selective and canagliflozin shows substantially lower selectivity [82,83,84]. These profiles are assay and species dependent and must be interpreted against local unbound concentrations. Blood–brain barrier penetration also requires compound-specific assessment; selectivity or lipophilicity alone cannot establish adequate unbound CNS exposure during therapeutic dosing. Off-target actions further complicate interpretation; canagliflozin can inhibit mitochondrial complex 1 and activate AMPK independently of SGLT2 in experimental systems [56]. Accordingly, central responses to one inhibitor should not be generalized to the class without comparable CNS exposure and target-validation data. Kim and colleagues subsequently examined hypothalamic AgRP and POMC regulation after intracerebroventricular administration of empagliflozin in high-fat-diet-fed mice. Empagliflozin was administered at 50 nM in 2 μL, followed by the assessment of hypothalamic AgRP and POMC expression. Complementary experiments in mHypoA hypothalamic cell lines evaluated neuropeptide transcription and FoxO1-related signaling over a broader concentration range [16]. The intracerebroventricular experiment reduces confounding from the canonical renal route, whereas the cellular studies demonstrate that hypothalamus-derived cells can respond to direct drug exposure. These results strengthen the evidence for central pharmacological capacity but do not demonstrate CNS SGLT2 dependence. The study did not use cell-specific SGLT2 deletion, transporter rescue, or a validated target-engagement assay. Moreover, transcriptional responses varied with empagliflozin concentration in the cell experiments, and the relationship between those concentrations and unbound hypothalamic exposure during clinical dosing remains unknown. Changes in FoxO1, AgRP, or POMC expression are therefore better described as responses to direct empagliflozin exposure rather than definitive consequences of neuronal SGLT2 inhibition. More broadly, intracerebroventricular administration shows that a compound has the capacity to influence the CNS after direct placement into the ventricular system. It does not establish that adequate concentrations reach the same cells after systemic treatment. Central administration studies should therefore be interpreted together with quantitative CNS pharmacokinetics, transporter selectivity, and genetic target-validation experiments.

### 4.3. Human Hypothalamic and Food-Cue Neuroimaging

Among the available translational studies, the randomized, double-blind phase 2 trial conducted by Kullmann and colleagues provides important evidence that systemic SGLT2 inhibitor therapy can modify human hypothalamic physiology. Participants with prediabetes received empagliflozin or placebo for 8 weeks, and hypothalamic insulin responsiveness was assessed using functional magnetic resonance imaging during intranasal insulin administration. Empagliflozin increased hypothalamic responsiveness to intranasal insulin, as assessed by insulin-induced changes in cerebral blood flow measured using functional MRI, and this increase was associated with reductions in fasting glucose and hepatic fat content [15]. The randomized design, human setting, and inclusion of a physiologically relevant insulin challenge make this strong evidence of treatment-associated central adaptation. The study did not, however, measure empagliflozin in cerebrospinal fluid or brain tissue, quantify unbound hypothalamic drug exposure, or demonstrate hypothalamic SGLT2 occupancy. Improved glycemia, altered insulin exposure, reduced hepatic fat, changes in circulating substrates, or attenuation of systemic inflammation could increase hypothalamic insulin responsiveness without direct drug action in the brain. Furthermore, the observed association between the hypothalamic response and peripheral metabolic outcomes does not establish the direction of causality. Human food-cue imaging studies provide complementary evidence from anatomically distinct regions. In participants with obesity and type 2 diabetes, dapagliflozin increased food-cue-related activation in the left putamen after 10 days, whereas this response was not maintained after 16 weeks. Combination treatment with exenatide produced different longer-term responses, including changes in the amygdala [20]. These results suggest that SGLT2 inhibition may transiently alter reward- or motivation-related neural responses. However, the principal reported regions did not include the hypothalamus, and blood-oxygen-level-dependent signals do not identify either the molecular target or whether the effect is direct. Human neuroimaging studies should therefore be regarded as evidence that systemic SGLT2 inhibitor treatment can modify CNS function, not as evidence that these drugs directly inhibit SGLT2 in human hypothalamic neurons. Studies combining neuroimaging with plasma and cerebrospinal fluid pharmacokinetics, metabolic phenotyping, and time-resolved measurements of hormones and ketones could help distinguish direct exposure from peripheral mediation.

### 4.4. Brainstem Neuronal Evidence

Potential central effects of SGLT inhibition may extend beyond hypothalamic circuits. Oshima and colleagues examined isolated brainstem-spinal cord preparations from newborn Wistar rats and directly superfused the tissue with inhibitors targeting SGLT2 or SGLT1. Pharmacological exposure hyperpolarized bulbospinal RVLM neurons and suppressed their firing activity. Immunohistochemical analyses also indicated SGLT-related immunoreactivity in the RVLM [19]. Because the preparation lacked an intact kidney and systemic circulation, the electrophysiological response shows that the neonatal brainstem preparation was directly sensitive to SGLT inhibition. Several limitations constrain the translation of these findings. First, the experiments were performed in newborn animals, whose transporter expression, blood–brain barrier properties, neuronal metabolism, and autonomic circuitry may differ from those in adults. Second, the RVLM is a brainstem sympathetic premotor region that has a central role in tonic and reflex control of sympathetic vasomotor activity, rather than a canonical hypothalamic nutrient-sensing nucleus [85]. Third, responses to both SGLT1- and SGLT2-directed inhibitors raise the possibility of overlapping transporter contributions. Fourth, immunoreactivity alone does not demonstrate transporter specificity or functional SGLT2-mediated glucose flux. Finally, superfusion directly exposes neural tissue to drug concentrations that may not occur in the adult RVLM after therapeutic oral dosing. These findings therefore indicate direct pharmacological sensitivity of an ex vivo central autonomic preparation and support a possible broader SGLT-sensitive neural network. They do not establish that direct inhibition of adult RVLM SGLT2 is responsible for sympathoinhibition during clinical SGLT2 inhibitor therapy. Reduced sympathetic activity after systemic treatment may instead result from, or be augmented by, altered renal afferent signaling, improved glycemia, changes in sodium or volume balance, reduced inflammation, or other cardiorenal adaptations. Taken together, the available literature demonstrates three distinct levels of evidence: central pharmacological capacity after direct CNS exposure, treatment-associated changes in human brain physiology after systemic administration, and ex vivo sensitivity of central autonomic neurons to SGLT inhibition. None of these findings alone establishes therapeutically relevant CNS SGLT2 target engagement. A direct mechanism will require convergence of quantitative unbound CNS pharmacokinetics, cell-specific functional target validation, and loss-of-function experiments that separate central from renal SGLT2 activity. Figure 2 summarizes this evidence framework, and Table 1 compares study size and exposure conditions while distinguishing direct experimental exposure from established CNS target engagement during therapeutic systemic treatment.

## 5. Mechanisms Linking SGLT2 Inhibition to Hypothalamic Regulation

### 5.1. AgRP/POMC and Energy-Sensing Pathways

The melanocortin system provides a plausible interface between urinary calorie loss and central energy compensation. AgRP/NPY and POMC neurons respond to changes in glucose availability, insulin, leptin, ghrelin, fatty acids, and cellular energy state and influence feeding, glucose metabolism, thermogenesis, and autonomic function through anatomically and functionally distinct downstream circuits [30,60,86]. In particular, α-melanocyte-stimulating hormone can regulate a rapidly acting ARC-to-PVH satiety circuit, illustrating that melanocortin signaling includes both acute neuronal and longer-term transcriptional responses [86]. Intracerebroventricular administration studies indicate that SGLT inhibitors can modify lateral hypothalamic AMPK activity, FoxO1-associated signaling, AgRP/POMC expression, and acute food intake [14,16]. These experiments demonstrate central pharmacological capacity but do not establish that the same circuits are engaged at clinically relevant unbound CNS concentrations after oral treatment. Moreover, the available evidence does not quantify how much AgRP/POMC pathways contribute to sustained body-weight regulation. Acute molecular activation, short-term feeding responses, and chronic energy-balance adaptation should therefore be considered distinct temporal and evidentiary levels.

### 5.2. Insulin and Leptin Sensitivity

Increased hypothalamic responsiveness to intranasal insulin after empagliflozin treatment is the strongest human evidence of treatment-associated central metabolic plasticity [15]. This finding is physiologically relevant because insulin signaling in AgRP-expressing neurons and other hypothalamic populations can influence hepatic glucose production and systemic glucose homeostasis [87,88]. However, the direction of causality remains uncertain. Reduced glycemic exposure, decreased hepatic fat, increased peripheral insulin sensitivity, altered circulating metabolites, or attenuation of inflammatory signaling could restore central insulin action; increased central insulin responsiveness could, in turn, reinforce peripheral metabolic control. Treatment studies also indicate changes in circulating adipokines. Enavogliflozin reduced leptin after adjustment for weight change in a 24-week placebo-controlled trial analysis, whereas the adjusted adiponectin difference was not significant [89]. In the 52 weeks BEYOND trial, dapagliflozin increased adiponectin relative to glimepiride alongside reductions in adiposity [90]. These changes do not establish increased hypothalamic leptin sensitivity, which requires functional assessment, such as leptin-induced STAT3 phosphorylation or neuronal responses [91].

### 5.3. Astrocytes and Hypothalamic Inflammation

Hypothalamic metabolic inflammation is spatially and temporally dynamic, involving neurons, astrocytes, microglia, tanycytes, endothelial cells, and other neurovascular components. In experimental obesity, reactive gliosis and inflammatory signaling can occur rapidly in the mediobasal hypothalamus and may subsequently interfere with neuronal insulin and leptin responsiveness [35,92]. Astrocytes contribute to glucose and lactate handling, neurotransmitter recycling, hormonal access, synaptic remodeling, and inflammatory signaling; consequently, changes in astrocyte markers should not be interpreted automatically as either beneficial or pathological. Direct cellular exposure to empagliflozin attenuated high-glucose-induced astrocyte activation and nuclear factor κB (NF-κB) signaling, thereby identifying a testable glial pathway [17]. These findings primarily demonstrate molecular anti-inflammatory responses in vitro, rather than restored astrocyte–neuron metabolic coupling or neuronal insulin/leptin responsiveness. Establishing a direct mechanism additionally requires adequate unbound hypothalamic exposure and evidence of astrocytic SGLT2 dependence.

### 5.4. Microglial Metabolism and Inflammatory Signaling

Microglia can detect nutrient excess and initiate or amplify hypothalamic inflammatory responses through cytokine production, metabolic reprogramming, and communication with astrocytes and neurons [34,93]. Experimental studies have shown that saturated-fat exposure can activate mediobasal hypothalamic microglia and impair neuronal function, establishing microglia as active mediators rather than passive markers of metabolic inflammation [93]. Systemic dapagliflozin reduced microglia-associated hypothalamic inflammation in mice with high-fat diet-induced obesity, with mTOR/HK2-related glucose metabolism implicated in the response [18]. Reduced microglial activation and inflammatory signaling do not establish restored glial–neuronal communication or neuronal metabolic regulation. Concurrent systemic metabolic changes also limit attribution to direct microglial SGLT2 inhibition. Functional neuronal measurements coupled with glia-specific perturbation are needed to establish this link.

### 5.5. Oxidative Stress and Mitochondrial Function

Mitochondrial substrate oxidation, morphology, and reactive oxygen species generation contribute to hypothalamic nutrient sensing. In POMC neurons, glucose sensing and systemic glucose regulation depend partly on intact mitochondrial dynamics, whereas the disruption of mitofusin- or DRP1-dependent remodeling can impair glucose or leptin responsiveness [94,95,96]. Reactive oxygen species may function as physiological signaling intermediates; therefore, a reduction in reactive oxygen species does not inherently indicate improved hypothalamic function. SGLT2 inhibitors have improved indices of oxidative and mitochondrial function in multiple peripheral and neural experimental models [97], but evidence obtained specifically in defined hypothalamic cell populations at clinically relevant unbound CNS concentrations remains limited. Redox and mitochondrial effects should therefore be treated as potential downstream modifiers rather than established primary mechanisms of central SGLT2 inhibition. Particular caution is required at high experimental concentrations, at which SGLT-independent mitochondrial actions may occur.

### 5.6. Ketone Bodies as Indirect Kidney-to-Brain Metabolic Signals

Ketogenesis is a prototypical indirect kidney-to-brain mechanism that does not require CNS penetration of the SGLT2 inhibitor itself. Renal glucose loss alters insulin-to-glucagon signaling and hepatic substrate utilization, thereby favoring fatty acid oxidation and ketone production. Circulating β-hydroxybutyrate crosses the blood–brain barrier through monocarboxylate transporters and can be oxidized by neurons and glia, although cerebral uptake depends on circulating concentration, transporter expression, developmental stage, and metabolic adaptation [74,98]. In addition to serving as an oxidative substrate, β-hydroxybutyrate can influence cellular redox state, histone modification, inflammatory signaling, and the NLRP3 inflammasome [74,75]. The relevance of these mechanisms depends on ketone concentration and tissue exposure. Clinical studies have reported modest, submillimolar circulating β-hydroxybutyrate concentrations during SGLT2 inhibitor treatment [99], whereas some mechanistic experiments used substantially higher concentrations, including 10 mM in NLRP3 assays [100]. These experimental effects cannot be assumed to occur at therapeutic exposure or in hypothalamic neurons. Circulating concentrations also do not establish local hypothalamic exposure or functional neuronal responses. Physiological ketone signaling should be distinguished from pathological ketone accumulation with metabolic acidosis, including SGLT2 inhibitor-associated euglycemic ketoacidosis [101]. Exposure-matched studies combining ketone measurements, isotope tracing, and neuronal functional assessments are needed to establish causality. Table 2 summarizes these candidate mechanisms, their proposed hypothalamic sites, and their principal limitations.

## 6. Autonomic Regulation and the Brain–Kidney Axis

The kidney and central nervous system communicate in both directions through renal sympathetic efferents, spinal renal sensory afferents, and circulating hormones and hemodynamic, inflammatory, and metabolic signals. Efferent renal sympathetic nerve activity regulates renin secretion, tubular sodium reabsorption, renal vascular resistance, and renal hemodynamics. Renal sensory neurons, whose cell bodies are located predominantly in thoracolumbar dorsal root ganglia, detect mechanical, chemical, ischemic, and inflammatory changes within the kidney and transmit this information to the dorsal horn of the spinal cord and ascending autonomic pathways [21,62,102,103]. The effects of renal afferent activation depend on context. Under physiological conditions, mechanosensory activation can engage inhibitory renorenal reflexes that reduce efferent renal sympathetic activity and facilitate natriuresis. In contrast, renal ischemia, inflammation, elevated intrarenal pressure, or tissue injury can generate excitatory afferent input that contributes to sustained central sympathetic activation. Experimental stimulation of renal afferent nerves activates catecholaminergic and non-catecholaminergic neurons in the medulla and modulates paraventricular hypothalamic neurons projecting to the RVLM, thereby providing anatomically plausible routes through which renal sensory information can influence central autonomic output [104,105]. Central processing of kidney-derived signals involves a distributed network rather than a single obligatory relay. Ascending renal sensory information can influence the paraventricular nucleus of the hypothalamus, RVLM, nucleus tractus solitarius, and other spinal, brainstem, and forebrain regions involved in cardiovascular and metabolic regulation. These regions integrate renal input with baroreceptor, visceral, endocrine, inflammatory, and nutrient-related signals and regulate sympathetic output to the kidney and sympathetic and parasympathetic output to other peripheral organs. The hypothalamus is therefore best regarded as an important component of a distributed autonomic network linking renal status to whole-body metabolism, rather than as an obligatory relay for all kidney-derived signals [13,61,102,103].

### 6.1. Evidence for Autonomic Modulation by SGLT2 Inhibitors

SGLT2 inhibitors produce modest reductions in office and ambulatory blood pressure, often without a compensatory increase in heart rate. A meta-analysis of randomized trials estimated an average reduction in 24 h ambulatory blood pressure of approximately 3.6/1.7 mm Hg, an effect comparable in magnitude to that of low-dose hydrochlorothiazide [106]. This hemodynamic profile has been interpreted as compatible with the attenuation of sympathetic activation. However, reductions in blood pressure or stability of heart rate are indirect autonomic indices and do not independently demonstrate sympathoinhibition. Studies directly measuring muscle sympathetic nerve activity (MSNA) provide a more nuanced assessment. In patients with type 2 diabetes, 4 days of empagliflozin treatment did not significantly change MSNA despite diuresis, weight loss, and numerical reductions in blood pressure [65]. Similarly, in a 6-week randomized comparison of empagliflozin and hydrochlorothiazide, neither treatment significantly altered unadjusted MSNA. After adjustment for treatment-related weight change, however, the relationship between volume loss and sympathetic activation was shifted toward lower MSNA with empagliflozin. These findings suggest that empagliflozin may attenuate the reflex sympathoexcitation that ordinarily accompanies sodium and volume loss, but they do not establish a generalized or direct sympatholytic effect [66]. Consistent with this uncertainty, a meta-analysis of four randomized trials found no significant change in heart rate variability indices, including the low-frequency/high-frequency ratio, standard deviation of normal-to-normal intervals (SDNN), and root mean square of successive differences (rMSSD) [107]. Direct nerve-recording studies in experimental animals similarly suggest that autonomic effects may depend on metabolic state and physiological challenge. In rats maintained on either standard chow or a high-fat diet, chronic dapagliflozin treatment did not consistently reduce basal peripheral sympathetic nerve activity but modified the sympathetic response to an intravenous glucose load [108]. Current evidence does not establish class-wide sympathoinhibition or localize treatment effects to central autonomic circuits. MSNA reflects sympathetic traffic to skeletal muscle vasculature, whereas heart rate variability reflects the modulation of sinus rhythm; neither directly measures cardiac postganglionic sympathetic activity. Peripheral ganglionic mechanisms also warrant distinction; in rats with type 2 diabetes, increased stellate ganglion PKC–MAPK14–ADAM17 signaling was associated with enhanced cardiac sympathetic neuronal excitability and nerve activity [109]. This finding illustrates peripheral autonomic dysfunction but does not demonstrate an effect of SGLT2 inhibitors on sympathetic ganglia. Treatment-associated autonomic changes should therefore be interpreted according to the organ, recording site, and physiological conditions examined.

### 6.2. Potential Central and Renal Mechanisms

**Figure 3:**
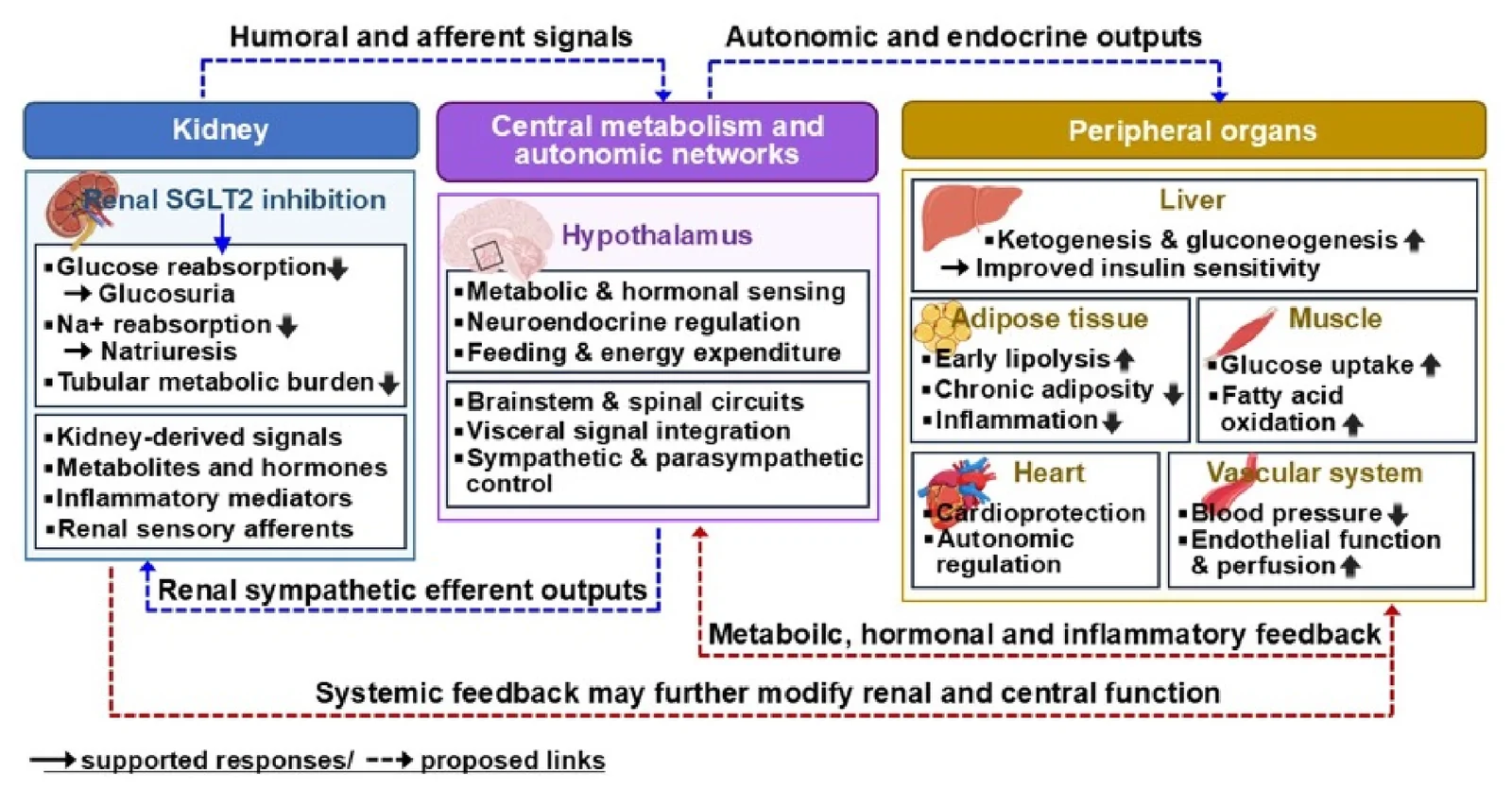
**Proposed brain–kidney–metabolism axis during sodium-glucose cotransporter 2 (SGLT2) inhibition.** Renal SGLT2 inhibition initiates glucosuria, natriuresis, and reduced proximal-tubule transport workload and may alter renal sensory signaling. Metabolic, hormonal, inflammatory, and afferent neural signals arising from renal and systemic adaptations may influence hypothalamic, brainstem, and spinal autonomic networks. These networks integrate metabolic and visceral information and provide neuroendocrine and autonomic outputs to the kidney and other peripheral organs. Liver, adipose tissue, skeletal muscle, heart, and vasculature provide reciprocal metabolic, hormonal, inflammatory, and neural signals. Solid arrows indicate physiological responses supported during SGLT2 inhibitor treatment, whereas dashed arrows indicate proposed mechanistic links. In particular, altered renal afferent signaling and its contribution to hypothalamic regulation remain unconfirmed during treatment. Upward and downward symbols indicate the direction of reported changes, which may vary by compound and physiological context. The schematic does not establish that central signaling mediates the cardiorenal benefits of SGLT2 inhibitors.

Autonomic modulation during SGLT2 inhibitor treatment could arise through several potentially convergent mechanisms. The RVLM experiments summarized in Section 4.4 demonstrate that selected brainstem neurons are pharmacologically sensitive to SGLT inhibition under ex vivo conditions. However, these experiments do not establish that therapeutically relevant unbound drug concentrations reach the adult RVLM after oral administration or that direct CNS target engagement mediates autonomic effects in vivo. Indirect mechanisms include changes in extracellular volume and baroreflex loading, reduced renal metabolic and inflammatory stress, improved glycemic and cardiorenal status, and altered renal sensory input. The renal-afferent hypothesis is physiologically plausible because pathological renal sensory signaling can sustain central sympathetic activation in kidney and cardiovascular disease [102,103,110]. Nevertheless, direct renal-afferent recordings, selective deafferentation experiments, or afferent-specific neural manipulations during SGLT2 inhibitor treatment are lacking. Altered renal afferent signaling and its contribution to hypothalamic regulation therefore remain proposed mechanisms during SGLT2 inhibition and are represented by dashed arrows in Figure 3. Circulating glucose, ketone bodies, insulin, glucagon, adipokines, cytokines, and sodium- or volume-related signals may also modify hypothalamic and brainstem activity without requiring CNS penetration of the inhibitor. These humoral and hemodynamic pathways provide alternative explanations for treatment-associated changes in autonomic regulation and may operate in parallel with renal neural signaling.

### 6.3. An Integrative Brain–Kidney–Metabolism Model

Overall, the available evidence supports a distributed and bidirectional model rather than a single central pathway. Renal SGLT2 inhibition modifies the metabolic, hemodynamic, inflammatory, and sensory information reaching central autonomic networks. Hypothalamic, brainstem, and spinal circuits may integrate these signals and adjust autonomic and neuroendocrine output to the kidney, cardiovascular system, liver, pancreas, adipose tissue, and skeletal muscle. Direct CNS SGLT inhibition, kidney-derived afferent signaling, and systemic humoral or hemodynamic effects may represent alternative but potentially convergent mechanisms. Their relative contributions remain unknown. Current human evidence is consistent with the attenuation of volume-loss-associated or stimulus-evoked sympathoexcitation under selected conditions, but it does not establish consistent suppression of basal sympathetic activity or demonstrate that central autonomic modulation is required for the cardiorenal benefits of SGLT2 inhibitors [65,66,107,108]. The mechanistic question should therefore extend beyond whether an SGLT2 inhibitor acts directly within the brain to include how renal SGLT2 inhibition reshapes the neural and humoral information entering central autonomic networks and the regulatory signals subsequently returned to the kidney and other metabolic organs. This proposed brain–kidney–metabolism axis is shown schematically in Figure 3.

## 7. Clinical and Translational Implications

### 7.1. Body Weight and Appetite Regulation

SGLT2 inhibitors generally produce less weight loss than predicted based on cumulative urinary energy loss, suggesting partial homeostatic compensation [11]. Mathematical modeling of longitudinal body-weight changes during canagliflozin treatment further suggested that increased energy intake substantially offsets the caloric deficit produced by glucosuria, although inferred energy intake should not be equated with directly measured appetite or food consumption [111]. Circulating hormone findings do not consistently explain this compensation. Apelin increased by approximately 18.3% after 6 months of dapagliflozin in an uncontrolled study of patients with type 2 diabetes and heart failure, whereas an analysis of a 4-week placebo-controlled empagliflozin trial in chronic SIAD found no significant change [112,113]. These findings do not establish an appetite-regulatory mechanism. In the 24-week SEESAW trial, empagliflozin produced no consistent changes in postprandial acylated ghrelin, PYY, GLP-1, or subjective appetite [114]. Thus, sustained activation of a single orexigenic pathway cannot be assumed to explain the weight response. Weight and appetite responses should therefore be interpreted as integrated adaptations to urinary energy loss and systemic metabolic changes, without assuming direct CNS SGLT2 inhibition.

### 7.2. Central Insulin Responsiveness and Type 2 Diabetes

The increase in hypothalamic responsiveness to intranasal insulin after empagliflozin treatment provides important evidence of treatment-associated central metabolic plasticity and may contribute to a reciprocal relationship between central and peripheral insulin action [15]. Central insulin signaling contributes to the regulation of appetite, hepatic glucose production, adipose tissue metabolism, and whole-body insulin sensitivity, although the magnitude and direction of these effects depend on metabolic phenotype and brain insulin sensitivity [88]. Baseline insulin resistance and adiposity may influence both central responsiveness and the extent of peripheral metabolic improvement. The hypothalamic findings in adults with prediabetes [15] therefore cannot be assumed to extend to advanced diabetes or severe obesity. Treatment duration also matters: dapagliflozin-associated food-cue responses differed between 10 days and 16 weeks [20]. These differences do not establish which patients benefit most or whether CNS changes precede or follow systemic improvement. Trials stratified by baseline insulin resistance and adiposity, with early and later assessments, would help distinguish transient responses from sustained adaptation.

### 7.3. Chronic Kidney Disease and Cardiovascular Disease

The cardiovascular and kidney benefits of SGLT2 inhibitors are established by large randomized outcome trials independently of any proposed CNS mechanism [2,3,4,115]. Nevertheless, the proposed brain–kidney framework may be particularly relevant to chronic kidney disease (CKD) and heart failure. Both disorders are characterized by neurohumoral activation, impaired autonomic regulation, inflammation, renal sensory signaling, and altered systemic and tissue substrate metabolism. Direct recordings have demonstrated increased sympathetic nerve activity in patients with chronic renal failure, including those with diseased native kidneys, supporting a contribution of kidney-derived neural signals to sympathetic activation [116,117]. Sympathetic activation is also associated with disease severity and adverse outcomes in heart failure [118]. These observations provide a physiological basis for investigating autonomic responses to SGLT2 inhibition but do not show that autonomic modulation mediates clinical benefit. Renal function may further modify CNS responses: reduced eGFR attenuates urinary glucose loss [119], while CKD and heart failure alter the neurohumoral and hemodynamic context. Whether these differences modify CNS responses remains untested. Comparing patients with preserved versus reduced renal function, while characterizing disease severity and baseline autonomic dysfunction, would help separate metabolic from cardiorenal influences without presuming central mediation of clinical benefit.

### 7.4. Neurological and Neurodegenerative Implications

Preclinical studies suggest that SGLT2 inhibitor treatment may influence neurotrophic and synaptic integrity. Enavogliflozin increased brain-derived neurotrophic factor (BDNF) expression and hippocampal postsynaptic density protein (PSD95) immunoreactivity in 5XFAD mice, alongside improved spatial memory [120]. Studies evaluating dapagliflozin containing regimens in diet-induced diabetic mice reported increased doublecortin and synaptophysin expression [121], while empagliflozin attenuated cortical neuronal loss assessed by NeuN staining in models incorporating diabetes and Alzheimer-related pathology [122]. These findings do not establish functional synaptic recovery or direct neuronal SGLT2 dependence, because concurrent metabolic and vascular improvements may contribute. Their relevance to hypothalamic regulation and therapeutic human exposure also remains uncertain. Clinical evidence on dementia risk is mixed. A Korean propensity score-matched cohort associated SGLT2 inhibitor initiation with lower dementia incidence than DPP-4 inhibitor initiation (HR, 0.65; 95% CI, 0.58–0.73) [123], whereas another cohort found no significant association with dementia (HR, 0.78; 95% CI, 0.55–1.12), despite lower mild cognitive impairment incidence [124]. Observational comparisons remain susceptible to residual confounding and outcome misclassification. A 2025 meta-analysis of randomized trials likewise found no significant reduction in dementia or cognitive impairment with SGLT2 inhibitors (OR, 1.20; 95% CI, 0.67–2.17), although sparse events and reliance on adverse-event reporting limit interpretation [125]. Dedicated trials incorporating standardized cognitive outcomes and neuronal or synaptic biomarkers are therefore required before dementia-preventive or disease-modifying effects can be established.

## 8. Knowledge Gaps and Future Directions

### 8.1. Quantifying CNS Exposure and Validating Target Engagement

Demonstration of therapeutic CNS target engagement requires compound-specific evidence showing that the pharmacologically active unbound drug reaches the relevant extracellular or intracellular compartment at concentrations sufficient to inhibit the proposed target. Total brain-to-plasma concentration ratios are insufficient because brain homogenates include residual vascular drug as well as protein-bound, membrane-associated, lysosomally sequestered, and otherwise pharmacologically unavailable fractions. At or near distribution equilibrium, studies should determine the unbound brain-to-plasma partition coefficient (*K*_p,uu,brain_) from unbound plasma and brain concentrations and report the methods used to estimate plasma and brain tissue binding [78,126,127]. Cerebral microdialysis can yield a relatively direct estimate of unbound extracellular drug concentration but requires compound-specific recovery calibration and is technically challenging in small hypothalamic and brainstem nuclei. Brain-slice or equilibrium-dialysis methods provide complementary estimates of tissue binding and intracellular distribution but do not independently establish extracellular target-site exposure. Whenever feasible, quantitative whole-body perfusion or correction for residual vascular volume should be used to minimize contamination of brain measurements by intravascular drug. Cerebrospinal fluid (CSF) concentrations may complement plasma and brain measurements but cannot be treated as an invariant surrogate for unbound hypothalamic or brainstem interstitial concentrations. CSF and regional brain interstitial fluid can differ because of distinct interfaces, active influx and efflux transporters, CSF production and turnover, intracellular sequestration, and distance from ventricular or subarachnoid compartments [78,128]. This distinction is especially important for the hypothalamus, where the median eminence, ARC-median eminence interface, deeper parenchyma, and ventricular surface have different vascular and barrier properties. Regional unbound exposure should be compared with the inhibitory potency of each compound against both SGLT2 and SGLT1 under physiologically relevant glucose, sodium, membrane-potential, and protein-binding conditions. Because transporter inhibition may be substrate- and assay-dependent, nominal in vitro IC_50_ values obtained under nonphysiological conditions cannot be interpreted as fixed thresholds for in vivo target engagement. Finally, exposure data should be linked to a proximal pharmacodynamic readout, such as sodium-dependent glucose transport, transporter-associated current, cellular substrate flux, or a validated target-dependent signaling response.

### 8.2. Establishing Functional and Cell-Specific SGLT2 Dependence

SGLT2 is strongly enriched in the renal proximal tubule, whereas its abundance, cellular distribution, and functional relevance in the CNS remain incompletely resolved. Reported SGLT2 immunoreactivity in choroid plexus epithelial and ependymal cells suggests candidate expression but does not establish functional expression in hypothalamic neurons or glia, nor does it demonstrate that these cells are exposed to pharmacologically active drug concentrations after oral administration [22]. Candidate CNS expression requires verification using orthogonal approaches that address the limitations of individual methods. Single-cell and single-nucleus RNA sequencing can resolve cellular heterogeneity within the ARC-median eminence complex but may fail to detect low-abundance transcripts because of limited sequencing depth and transcript dropout [29]. Absence of *Slc5a2* reads in an individual dataset is therefore not definitive evidence of absence. Conversely, detection in a small number of cells requires evaluation for ambient RNA contamination, doublets, batch effects, regional sampling, and species differences. Transcriptomic findings should be combined with spatial transcriptomics, RNAscope or comparable in situ hybridization, quantitative targeted proteomics, and immunohistochemistry using antibodies validated in *Slc5a2*-null tissue. General antibody-validation principles support the use of genetic controls, orthogonal methods, independent antibodies, and tagged-protein strategies rather than reliance on immunoreactivity alone [129]. Functional relevance should then be tested using sodium-dependent glucose transport assays, transporter-associated electrophysiological currents, isotope-tracer flux, or genetically encoded glucose and sodium sensors. Once functional expression has been demonstrated, adult-inducible and region- or cell-specific deletion can target candidate AgRP, POMC, astrocytic, microglial, tanycytic, choroid plexus, ependymal, or brainstem populations. Adult induction is preferable to constitutive deletion because it reduces the likelihood of developmental compensation. Efficient and selective recombination requires verification at the transcript, protein, and functional levels. Loss of a systemic drug response after CNS-specific *Slc5a2* deletion, despite preserved renal SGLT2 expression, glucosuria, natriuresis, and systemic drug exposure, would support direct CNS SGLT2 dependence. Preserved responses would favor indirect systemic mediation, incomplete deletion, or an alternative molecular target. Because several inhibitors show lower selectivity for SGLT2 over SGLT1 at sufficiently high concentrations, parallel assessment of *Slc5a1* dependence and SGLT-independent effects should inform experimental interpretation.

### 8.3. Separating Renal, Humoral, and Neural Mechanisms

Distinguishing direct CNS pharmacology from renal and systemic mediation requires factorial and convergent experimental designs. Relevant comparisons should include clinically matched systemic drug exposure, verified local CNS exposure, adult-inducible proximal-tubule-specific *Slc5a2* deletion, and experimentally matched glucosuria or urinary energy loss. Kidney-restricted inhibitors or compounds with similar renal potency but different CNS exposure could provide another pharmacological strategy, provided that renal exposure, selectivity, and systemic metabolic effects are comparable. Pair-feeding and sodium-, fluid-, glucose-, insulin-, glucagon-, and ketone-matched controls can help isolate selected downstream consequences of SGLT2 inhibition. However, no single matching paradigm reproduces the complete renal perturbation, and extensive matching can itself introduce nonphysiological interventions. These controls should therefore be used as complementary contrasts rather than complete substitutes for renal SGLT2 inhibition. The contribution of ketone signaling could be tested through pharmacological inhibition or genetic disruption of ketogenesis combined with controlled β-hydroxybutyrate replacement. Demonstration of necessity would require loss of the central response when ketone production or brain ketone utilization is prevented, whereas demonstration of sufficiency would require reproduction of the response by matched ketone exposure in the absence of SGLT2 inhibition. Because interventions affecting ketogenesis may also change lipid flux, hepatic energy metabolism, acid–base balance, and hormone concentrations, these variables should be measured explicitly. Renal sensory signaling also requires direct investigation. Conventional renal denervation disrupts both afferent sensory and efferent sympathetic fibers and therefore cannot determine the direction of neural transmission. Periaxonal capsaicin enables preferential ablation of renal sensory afferents while preserving renal efferent nerves [130]. However, this approach preferentially targets capsaicin-sensitive, TRPV1-expressing sensory neurons and may not eliminate all renal afferent populations. Verification should document loss of renal sensory markers or evoked afferent responses, preservation of efferent function, and assessment of possible reinnervation during chronic studies. Selective deafferentation or molecularly defined sensory-neuron manipulation should be combined with direct renal afferent and efferent nerve recordings, anatomical tracing, electrophysiology, and simultaneous profiling of circulating metabolites, hormones, and cytokines. Abolition of a hypothalamic or brainstem response after selective renal deafferentation, with preserved renal glucosuria and systemic metabolic changes, would support neural transmission. Persistence after deafferentation, together with reproduction by controlled metabolite or hormone replacement, would favor humoral mediation. Neither result alone would exclude parallel or redundant signaling pathways.

### 8.4. Resolving Anatomical, Temporal, and Human Heterogeneity

CNS exposure and physiological responses cannot be assumed to be spatially uniform. The fenestrated capillaries of the median eminence, the tanycyte-regulated ARC-median eminence interface, the deeper hypothalamic parenchyma, ventricular surfaces, and brainstem nuclei differ in vascular permeability, transporter expression, extracellular geometry, and access to circulating molecules. Fasting itself can induce tanycyte- and VEGF-A-dependent remodeling of the blood–hypothalamus barrier, demonstrating that access of circulating signals to the ARC is physiologically dynamic [31]. High permeability within the median eminence cannot therefore be generalized to the entire ARC or to deeper hypothalamic structures. Diabetes, obesity, CKD, heart failure, aging, biological sex, and concomitant medication may also modify neurovascular integrity, inflammatory state, renal function, systemic drug exposure, and autonomic tone. Sex hormones and sex-dependent immune and vascular mechanisms can influence blood–brain barrier function, supporting adequately powered sex-stratified analyses rather than statistical adjustment for sex alone [131]. Renal impairment may also alter plasma protein binding, transporter activity, metabolite accumulation, and systemic pharmacokinetics without corresponding changes in unbound regional CNS exposure. Acute intracerebroventricular dosing, short-term systemic administration, early neuroimaging, and chronic clinical treatment interrogate different exposure compartments and phases of adaptation. Longitudinal studies should prespecify acute, adaptive, and chronic time points and align compound-specific plasma and CNS exposure with neuronal, glial, metabolic, autonomic, and behavioral measurements. Washout or rechallenge phases could help distinguish persistent adaptation from responses dependent on continuous drug exposure. Human protocols could combine hypothalamic functional MRI, intranasal insulin challenge, autonomic phenotyping, continuous glucose monitoring, indirect calorimetry, objective food-intake assessment, body composition, and metabolomic or hormonal profiling. Active comparators should be selected based on the specific causal contrast, for example, matching glycemic improvement, natriuresis, weight loss, ketone exposure, or blood-pressure reduction, because no single comparator can match all systemic consequences of SGLT2 inhibition. Whenever possible, population pharmacokinetic modeling and plasma or CSF sampling should be prospectively linked to imaging and physiological outcomes to establish an exposure–response relationship. Studies should also specify in advance whether the primary objective is to detect a central physiological response, establish direct CNS SGLT2 target engagement, or determine mediation of a systemic clinical effect. These questions require different designs and cannot be inferred from one another. Table 3 summarizes these knowledge gaps and a proposed experimental roadmap for resolving them.

## 9. Conclusions

SGLT2 inhibitors exert their best-established pharmacological action in the renal proximal tubule, where inhibition of glucose and sodium reabsorption initiates a coordinated series of metabolic, endocrine, hemodynamic, inflammatory, and potentially neural adaptations. Emerging experimental and human evidence indicates that SGLT2 inhibitor treatment can alter hypothalamic and brainstem physiology, including pathways related to nutrient sensing, insulin responsiveness, feeding regulation, glial inflammation, and autonomic activity. However, these observations do not establish a unified direct CNS mechanism or demonstrate that central signaling mediates the established cardiorenal benefits of this drug class. The available evidence should instead be interpreted based on the level at which it was obtained. Intracerebroventricular administration, direct cellular exposure, and ex vivo neural preparations demonstrate the capacity of SGLT-sensitive central pathways to respond to pharmacological manipulation. In contrast, systemic animal studies and human neuroimaging demonstrate treatment-associated CNS adaptation without establishing therapeutically relevant CNS exposure, cell-specific SGLT2 engagement, or causal mediation. Central pharmacological capacity, therapeutic target engagement, and physiological mediation therefore remain distinct claims and should not be used interchangeably. Within this framework, the proposed brain–kidney–metabolism axis identifies hypothalamic, brainstem, and spinal autonomic networks as candidate integrators of kidney-derived metabolic, hormonal, inflammatory, hemodynamic, and sensory signals. Renal glucosuria and natriuresis may alter circulating substrates, insulin-to-glucagon balance, ketone availability, adiposity-related hormones, inflammatory tone, volume status, and renal afferent activity. These signals may converge on central circuits that adjust behavioral, neuroendocrine, and autonomic outputs to the kidney, liver, pancreas, adipose tissue, skeletal muscle, cardiovascular system, and other peripheral organs. Feedback from these organs may in turn modify renal and central function, creating a dynamic and bidirectional regulatory network. This integrative model should be regarded as complementary to, not a replacement for, established renal tubular, hemodynamic, cardiac, vascular, and tissue-specific mechanisms of SGLT2 inhibition. Central responses may represent causal mediators of selected systemic effects, compensatory adaptations to renal energy and sodium loss, parallel consequences of peripheral metabolic improvement, or pharmacological epiphenomena. Their relative contributions are likely to vary according to the specific compound, dose, treatment duration, metabolic state, disease phenotype, age, and sex. Resolving these possibilities will require compound-specific measurements of unbound CNS exposure, orthogonal validation of functional and cell-specific SGLT2 expression, and proximal pharmacodynamic evidence of target engagement. Adult-inducible cell- and organ-specific genetic models, kidney-restricted pharmacological approaches, selective manipulation of renal sensory pathways, and experiments that separately control glucosuria, natriuresis, ketone availability, caloric loss, and systemic hormonal changes are essential to establish causality. Longitudinal human studies integrating neuroimaging, metabolic flux measurements, autonomic phenotyping, objective assessment of energy intake and expenditure, and exposure–response analysis are equally important for determining clinical relevance. Ultimately, the central question is not only whether SGLT2 inhibitors can act within the brain, but how renal SGLT2 inhibition reshapes the information exchanged among the kidney, central metabolic networks, and peripheral organs. Defining this communication could provide a more complete physiological account of whole-body adaptation to SGLT2 inhibition while identifying new therapeutic opportunities for disorders characterized by impaired brain-periphery metabolic and autonomic regulation.

## Figures and Tables

**Table 1:** Key studies evaluating CNS-associated responses to SGLT inhibition and the strength of evidence for a direct central mechanism.

Ref.	Drug/Model; Sample Size	Route; Dose; Duration	Main Endpoint/Finding	Interpretation of Direct CNS Evidence
**Takeda** **et al., 2021 [14]**	Tofogliflozin, dapagliflozin, empagliflozin/rats; n = 7–18/group, depending on the experiment	Single intracerebroventricular (i.c.v) infusion; 0.52 μmol/3 μL	Increased food intake and hypothalamic AMPK phosphorylation/c-Fos expression following central SGLT2 inhibitor	Supports central pharmacological capacity; relevance to therapeutic systemic exposure remains uncertain
**Kullmann et al., 2022 [15]**	Empagliflozin/ overweight-obese adults with prediabetes; 42 randomized, 40 completed	Oral, 25 mg once daily; 8 weeks	Increased hypothalamic insulin responsiveness assessed by fMRI following intranasal insulin administration	Demonstrates a treatment-associated CNS response; CNS drug exposure and direct target engagement were not established
**Ruiten** **et al., 2022 [20]**	Dapagliflozin ± exenatide; adults with obesity and T2DM; fMRI cohort: n = 64 across four groups	Oral dapagliflozin, 10 mg once daily; 16 weeks (assessments at 10 days and 16 weeks)	Increased food-cue-induced left putamen activation with dapagliflozin at 10 days, but not 16 weeks; combination therapy reduced right amygdala activation at 16 weeks	Demonstrates altered food-cue responses during systemic treatment; CNS drug exposure and direct SGLT2 target engagement were not established
**Kim et al., 2024 [16]**	Empagliflozin/male mice; i.c.v-vehicle/EMPA-n = 8/11 HFD-induced mice; oral-vehicle/EMPA- n = 5/5	i.c.v., 50 nM in 2 μL at 1 μL/min; assessment over 24 h Oral, 10 mg/kg/day; 3 weeks	Modified hypothalamic AgRP/POMC expression with changes in FoxO1-associated transcriptional signaling	Supports responses to direct central and cellular exposure; SGLT2 dependence and relevance to therapeutic CNS exposure remain unestablished
**Kim et al., 2024 [17]**	Empagliflozin/male mice; n = 5/group	Oral, 10 mg/kg/day; 3 weeks	Attenuated HFD-induced hypothalamic astrocyte activation and inflammatory cytokine expression	CNS-associated effects after systemic treatment; direct action cannot be separated from peripheral metabolic effects
**Chen et al., 2024 [18]**	Dapagliflozin; female mice; chow/chow + DAPA/HFD/HFD + DAPA: n = 11/11/15/16	Dietary administration, 1 mg/kg; 22 weeks.	Reduced hypothalamic microglial activation and NLRP3/IL-1β expression, with improved ovulatory function.	CNS-associated effects after systemic treatment; direct CNS action cannot be separated from peripheral metabolic effects.
**Lee et al., 2026 [46]**	Dapagliflozin; adults with T2DM; 42 randomized, 34 completed (n = 17/group). Empagliflozin; male OLETF diabetic rats; n = 5–6	Human: Oral, 10 mg once daily; 24 weeks; sitagliptin comparator, 100 mg/day Rats: Drinking water, 10 mg/kg/day; 12 weeks.	Reduced body weight and leptin without increased food intake or appetite in humans; transient early hyperphagia without sustained changes in food intake or hypothalamic AgRP/POMC expression in rats.	Supports temporal adaptation during systemic treatment; direct CNS action and target engagement remain unestablished.

Dose and concentration refer to administered amounts or nominal experimental concentrations, not measured unbound CNS exposure. Intracerebroventricular administration and direct cellular or tissue exposure bypass blood–brain barrier delivery constraints and should not be assumed to reproduce exposure during therapeutic oral dosing. Sample sizes refer to the indicated experiment or analysis.

**Table 2:** Candidate pathways linking renal SGLT2 inhibition to central metabolic regulation and their evidentiary limitations.

Mechanism	Proposed CNS Site	Direct/Indirect Pathway	Major Limitation
AgRP/POMC regulation [16]	Arcuate nucleus (ARC)	Direct-exposure evidence plus systemic mediation	Physiological relevance of central experimental dose
AMPK energy sensing [14]	Lateral hypothalamus/ARC	Direct-exposure evidence plus indirect energetic effects	Acute molecular response may not predict chronic feeding
Hypothalamic insulin sensitivity [15]	Hypothalamic insulin network	Mixed/indirect likely	Direct drug engagement not shown
Astrocyte NF-κB signaling [17]	Hypothalamic astrocytes	Potential direct	Therapeutic CNS concentration unknown
Microglial inflammation [18]	Mediobasal hypothalamus	Direct cellular exposure plus systemic mediation; indirect dominant in vivo	Strong systemic metabolic confounding
Ketone utilization/signaling [73,100]	Neurons and glia	Proposed indirect	Causality not demonstrated
Oxidative stress/mitochondrial function [97]	Neurons/glia	Direct/indirect contributions unresolved	Much evidence derives from non-hypothalamic tissues
Sympathetic regulation [19,65]	Hypothalamus/RVLM	Direct + indirect	SGLT1 involvement and limited human mechanistic data
Leptin signaling [89]	Arcuate nucleus (ARC)	Predominantly indirect	Changes in leptin concentration do not establish increased sensitivity

**Table 3:** Critical knowledge gaps and experiments required to distinguish direct CNS pharmacology from renal or systemic mediation.

Knowledge Gap	Why Current Evidence Is Insufficient	Recommended Experiment	Decisive Outcome
Therapeutic CNS penetration	Total brain concentrations do not define target exposure	Unbound plasma/CSF/brain microdialysis with LC-MS/MS	Regional unbound CNS exposure sufficient for target inhibition
Functional CNS SGLT2 expression	mRNA or immunostaining alone is insufficient	RNAscope + spatial omics + functional glucose transport assays	Cell-specific functional SGLT2 activity
Neuronal target dependency	Pharmacological response may be off-target	AgRP-, POMC-, astrocyte-, microglia-specific *Slc5a2* knockout	Loss of SGLT2i-induced CNS phenotype
Direct vs. renal-mediated effects	Systemic therapy simultaneously changes multiple metabolites	Oral vs. ICV treatment + kidney-specific knockout + glucosuria-matched controls	Dissociation of CNS drug effect from renal metabolic effect
Role of ketones	Association does not prove mediation	Blockade of ketogenesis and controlled beta-hydroxybutyrate replacement	Loss/rescue of central phenotype
Kidney-to-brain neural signaling	Renal afferent contribution poorly defined	Selective renal deafferentation, tracing, direct nerve recording, and afferent-specific manipulation	Attenuation of hypothalamic/RVLM response
Acute vs. chronic adaptation	Experiments examine different time scales	Longitudinal CNS and metabolic phenotyping	Time-resolved transition from acute compensation to chronic adaptation
Human relevance	Most mechanistic evidence remains preclinical	fMRI/PET + metabolic phenotyping + pharmacokinetic studies	Reproducible CNS change linked to defined systemic mediator

## Data Availability

No new data were created or analyzed in this study. Data sharing is not applicable to this article.

## Abbreviations

The following abbreviations are used in this manuscript:

ADP	Adenosine diphosphate
AgRP	Agouti-related peptide
Akt	Protein kinase B
AMP	Adenosine monophosphate
AMPK	AMP-activated protein kinase
ARC	Arcuate nucleus
ATP	Adenosine triphosphate
BBB	Blood–brain barrier
CART	Cocaine- and amphetamine-regulated transcript
CKD	Chronic kidney disease
CNS	Central nervous system
CSF	Cerebrospinal fluid
DRP1	Dynamin-related protein 1
fMRI	Functional magnetic resonance imaging
FoxO1	Forkhead box O1
GLP-1	Glucagon-like peptide-1
HFD	High-fat diet
HK2	Hexokinase 2
HPA	Hypothalamic–pituitary–adrenal
IC_50_	Half-maximal inhibitory concentration
ICV/i.c.v.	Intracerebroventricular
LC-MS/MS	Liquid chromatography-tandem mass spectrometry
mRNA	Messenger RNA
MRI	Magnetic resonance imaging
MSNA	Muscle sympathetic nerve activity
mTOR	Mechanistic target of rapamycin
mTORC1	Mechanistic target of rapamycin complex 1
NF-κB	Nuclear factor kappa B
NLRP3	NLR family pyrin domain containing 3
NPY	Neuropeptide Y
NTS	Nucleus tractus solitarius
PET	Positron emission tomography
PI3K	Phosphoinositide 3-kinase
PNS	Parasympathetic nervous system
POMC	Pro-opiomelanocortin
PVH	Paraventricular hypothalamus
PYY	Peptide YY
RNA	Ribonucleic acid
rMSSD	Root mean square of successive differences
RVLM	Rostral ventrolateral medulla
SDNN	Standard deviation of normal-to-normal intervals
SGLT	Sodium-glucose cotransporter
SGLT1	Sodium-glucose cotransporter 1
SGLT2	Sodium-glucose cotransporter 2
SGLT2i	Sodium-glucose cotransporter 2 inhibitor
SGLT3	Sodium-glucose cotransporter 3
*SLC5A2/Slc5a2*	Solute carrier family 5 member 2
*Slc5a1*	Solute carrier family 5 member 1
SNS	Sympathetic nervous system
STAT3	Signal transducer and activator of transcription 3
T2DM	Type 2 diabetes mellitus
TRPV1	Transient receptor potential vanilloid 1
VEGF-A	Vascular endothelial growth factor A
VMH	Ventromedial hypothalamus
